# Rapid Transfer Alignment of MEMS SINS Based on Adaptive Incremental Kalman Filter

**DOI:** 10.3390/s17010152

**Published:** 2017-01-14

**Authors:** Hairong Chu, Tingting Sun, Baiqiang Zhang, Hongwei Zhang, Yang Chen

**Affiliations:** 1 Changchun Institute of Optics, Fine Mechanics and Physics, Chinese Academy of Sciences, Changchun 130033, China; chuhr@ciomp.ac.cn (H.C.); xgdzbq@163.com (B.Z.); zhanghw135@163.com (H.Z.); chenyang4688@163.com (Y.C.); 2 University of Chinese Academy of Sciences, Beijing 10049, China

**Keywords:** MEMS IMU, transfer alignment, adaptive incremental Kalman filter, information fusion

## Abstract

In airborne MEMS SINS transfer alignment, the error of MEMS IMU is highly environment-dependent and the parameters of the system model are also uncertain, which may lead to large error and bad convergence of the Kalman filter. In order to solve this problem, an improved adaptive incremental Kalman filter (AIKF) algorithm is proposed. First, the model of SINS transfer alignment is defined based on the “Velocity and Attitude” matching method. Then the detailed algorithm progress of AIKF and its recurrence formulas are presented. The performance and calculation amount of AKF and AIKF are also compared. Finally, a simulation test is designed to verify the accuracy and the rapidity of the AIKF algorithm by comparing it with KF and AKF. The results show that the AIKF algorithm has better estimation accuracy and shorter convergence time, especially for the bias of the gyroscope and the accelerometer, which can meet the accuracy and rapidity requirement of transfer alignment.

## 1. Introduction

**Figure 1:**
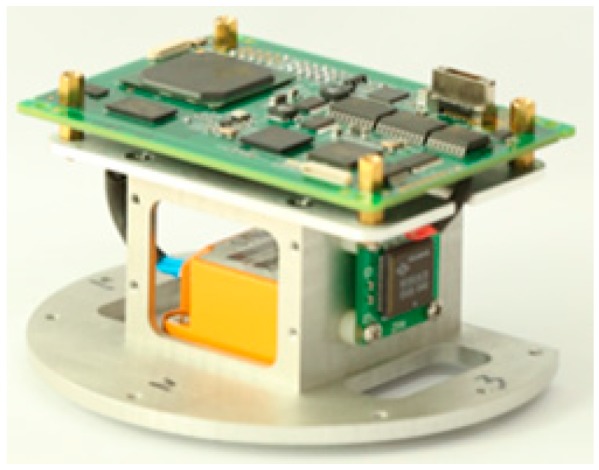
MEMS inertial measurement units.

Mirco-Electronical Mechanical System Inertial Measurement Unit (MEMS IMU), as shown in Figure 1, has been widely used in the Strapdown Inertial Navigation Systems (SINS) field in recent years because it has low cost, small size, and low power consumption [1]. In airborne MEMS SINS application, before the operation of the slaved SINS, it must be initially aligned using the master SINS and Kalman filter [2]. However, MEMS IMU has poor bias repeatability and bias drift level owing to the low-cost fabrication process, which may lead to large attitude error of the SINS in long time navigation cases [3,4]. A gyroscope with hundreds of deg/h of bias may lead to large nonlinear error in the progress of SINS transfer alignment. In the meantime, the uncertainty of noise, the lever arm effect, elastic distortion, and other disturbance during some violent maneuvers in practical application will make the transfer alignment more complex [5]. Besides, in airborne transfer alignment, the system errors of the measurement equation are usually unknown and the model parameters are also uncertain, which may lead to large error and bad convergence of the Kalman filter.

In order to improve the performance of the filter, many researchers are involved in developing improved filtering algorithms. In [6] an H∞ filter and Unscented Transformation (UT) algorithm are introduced. When applied to a nonlinear model, a model with colored noise, or an unmatched model, H∞ filter is still robust. An adaptive Unscented Particle filter is introduced in [7] to solve the initial alignment of SINS with large misalignment. In [8], a robust adaptive filter algorithm based on local observability analysis is developed for rapid transfer alignment. Simulation results indicate that the proposed method has better performance and shorter alignment time. In [9], a new multiple fading factors Kalman filtering algorithm is presented to solve the problem that the Kalman filter cannot give the optimal solution when the accurate system model and stochastic information are unknown. It uses the innovation sequence to compute multiple fading factors to scale the predicted covariance matrix. A similar conclusion can be drawn in [10]. By carefully designing a filter, we can develop an algorithm that is less sensitive to uncertain noise and has a better estimation effect, which is important for transfer alignment. From these references, we can see that the proper filter algorithm for MEMS SINS transfer alignment should be more robust and adaptive. This is the main idea of our research.

Based on these studies on the alignment algorithm, we proposed an improved transfer alignment algorithm based on AIKF. The paper is organized as follows. First, the model of SINS transfer alignment is defined, which includes the SINS mechanized arrangement, the SINS error model, the flexural deflection error model, and the state and measurement equations of the transfer alignment. Then, the algorithm of AIKF is presented with detailed description. The calculation amount and the performance of AKF and AIKF are also compared. Finally, a digital simulation test based on MATLAB is designed. The results are compared with traditional methods to test its performance.

## 2. Model of SINS Transfer Alignment

### 2.1. SINS Mechanized Arrangement

The local geographical coordinate is chosen as the navigation reference frame (*n*-frame), and the SINS mechanized arrangement equations are:

(1) $$\{\begin{array}{c} {\dot{C}}_{b}^{n}=C_{b}^{n}\left[\omega_{ib}^{b}\times \right]-\left[\omega_{in}^{n}\times \right]C_{b}^{n}\\ {\dot{V}}_{e}^{n}=f^{n}-\left(2\omega_{ie}^{n}+\omega_{en}^{n}\right)\times V_{e}^{n}+g^{n} \end{array}$$

where $C_{b}^{n}$ is the direction cosine matrix of the body frame with respect to the *n*-frame. $V_{e}^{n}$ is the velocity of the vehicle with respect to the earth in the navigation frame. $f^{n}$ is the specific force of the vehicle. $\omega_{in}^{n}$ is the rotation angular rate of *n*-frame with respect to the inertial frame (*i*-frame). $\omega_{ie}^{n}$ is the earth rotation angular rate. $\omega_{en}^{n}$ is the rotation angular rate of *n*-frame with respect to the earth. $g^{n}$ is the local gravity acceleration in *n*-frame [11].

### 2.2. SINS Error Model

The attitude error equation of SINS is,

(2) $$\dot{\varphi^{n}}=-\omega_{in}^{n}\times \varphi^{n}+\delta \omega_{in}^{n}-C_{b}^{n}\varepsilon^{b}$$

where φ is the attitude error angle of the slave SINS between the calculated *n*-frame and the real *n*-frame. $\omega_{in}^{n}$ is the rotation angular rate of *n*-frame with respect to the *i*-frame, and $\delta \omega_{in}^{n}$ is the error of $\omega_{in}^{n}$. $C_{b}^{n}$ is the direction cosine matrix of the body frame with respect to the *n*-frame. $\varepsilon^{b}$ is the equivalent bias drift error of the gyroscope.

The velocity error equation of SINS is,

(3) $$\dot{\delta V_{e}^{n}}=f^{n}\times \varphi^{n}-\left(2\omega_{ie}^{n}+\omega_{en}^{n}\right)\times \delta V_{e}^{n}+V_{e}^{n}\times \left(2\delta \omega_{ie}^{n}+\delta \omega_{en}^{n}\right)+C_{b}^{n}\nabla^{b}$$

where $\delta V_{e}^{n}$ is the velocity error of the vehicle in *n*-frame. $\nabla^{b}$ is the equivalent bias drift error of the accelerometer. $\delta \omega_{ie}^{n}$ and $\delta \omega_{en}^{n}$ are the angular rate error of $\omega_{ie}^{n}$ and $\omega_{en}^{n}$, respectively [12].

### 2.3. Flexural Deflection Error Model

When the vehicle is mounted on the aircraft, the airframe may suffer from flexural deflection caused by the disturbance of airflow or some maneuvers such as turns, climbs, and swings. This problem will lead to instability or large error of the alignment. Therefore, it is necessary to take the dynamic flexural deflection into consideration during the process of the transfer alignment [13].

The flexural deflection of the aircraft tends to be a stochastic progress, which can be modeled as a second-order Markov progress actuated by white noise. Supposing the deflection between the main SINS and the slave SINS in three axes are independent, the flexural deflection can be described as:

(4) $${\ddot{\theta }}_{i}+2\beta_{i}{\dot{\theta }}_{i}+\beta_{i}^{2}\theta_{i}=\eta_{i}\left(i=x,y,z\right)$$

where $\theta ={\left[\begin{array}{ccc} \theta_{x} & \theta_{y} & \theta_{z} \end{array}\right]}^{T}$ is the angular vector of the flexural deflection and its variance is $\sigma_{\theta }={\left[\begin{array}{ccc} \sigma_{\theta_{x}} & \sigma_{\theta_{y}} & \sigma_{\theta_{z}} \end{array}\right]}^{T}$. $\eta ={\left[\begin{array}{ccc} \eta_{x} & \eta_{y} & \eta_{z} \end{array}\right]}^{T}$ is the white noise with PSD of $Q_{\eta }={\left[\begin{array}{ccc} Q_{\eta_{x}} & Q_{\eta_{y}} & Q_{\eta_{z}} \end{array}\right]}^{T}$, which means $\eta \sim N\left(0,Q_{\eta }\right)$. $\beta ={\left[\begin{array}{ccc} \beta_{x} & \beta_{y} & \beta_{z} \end{array}\right]}^{T}$ is a constant value. The relationship between $Q_{\eta }$, $\sigma_{\theta }$, and β can be shown as $Q_{\eta i}=4\beta_{i}^{2}\sigma_{i}^{2}$. $\tau_{i}$ is the correlation time of the stochastic progress. The relationship between $\beta_{i}$ and $\tau_{i}$ can be shown as $\beta_{i}=2.146/\tau_{i}$.

Suppose that $\theta_{f}={\left[\begin{array}{ccc} \theta_{fx} & \theta_{fy} & \theta_{fz} \end{array}\right]}^{T}$ is the flexural deflection in the body frame (*b*-frame) and $\omega_{f}={\left[\begin{array}{ccc} \omega_{fx} & \omega_{fy} & \omega_{fz} \end{array}\right]}^{T}$ is the deflection angular rate of slave SINS with respect to the main SINS. Then we have:

(5) $${\dot{\theta }}_{f}=\omega_{f}$$

(6) $$\{\begin{array}{c} {\dot{\omega }}_{fx}=-\beta_{x}^{2}\theta_{fx}-2\beta_{x}\theta_{fx}+w_{\theta_{x}}\\ {\dot{\omega }}_{fy}=-\beta_{y}^{2}\theta_{fy}-2\beta_{y}\theta_{fy}+w_{\theta_{y}}\\ {\dot{\omega }}_{fz}=-\beta_{z}^{2}\theta_{fz}-2\beta_{z}\theta_{fz}+w_{\theta_{z}} \end{array}$$

where $w_{\theta_{x}}$, $w_{\theta_{y}}$, and $w_{\theta_{z}}$ are the white noise. $\beta_{i}=2.146/\tau_{i}$, where $\tau_{i}$ is the correlation time of the three axes.

The equations above can be rewritten as:

(7) $$\left[\begin{array}{c} {\dot{\theta }}_{f}\\ {\dot{\omega }}_{f} \end{array}\right]=\left[\begin{array}{cc} 0 & I\\ A_{1} & A_{2} \end{array}\right]\left[\begin{array}{c} \theta_{f}\\ \omega_{f} \end{array}\right]+\left[\begin{array}{c} 0\\ \eta \end{array}\right]$$

where $A_{1}=\left[\begin{array}{ccc} -\beta_{x}^{2} & 0 & 0\\ 0 & -\beta_{y}^{2} & 0\\ 0 & 0 & -\beta_{z}^{2} \end{array}\right]$, $A_{2}=\left[\begin{array}{ccc} -2\beta_{x} & 0 & 0\\ 0 & -2\beta_{y} & 0\\ 0 & 0 & -2\beta_{z} \end{array}\right]$, $\eta =\left[\begin{array}{c} w_{\theta_{x}}\\ w_{\theta_{y}}\\ w_{\theta_{z}} \end{array}\right]$

### 2.4. State and Measurement Equations of the Transfer Alignment

In 1989, Kain and Cloutier proposed the “Velocity and Attitude” matching method, which combined the advantage of velocity matching with attitude matching [14]. This algorithm has better alignment accuracy and costs less alignment time so it has been used in practical applications. Thus, in this paper we propose a new filter algorithm based on this matching method.

Based on the study of SINS error model, IMU error, misalignment error, and flexural deflection, we select the attitude error of the slave SINS, velocity error, bias drift of gyroscope and accelerometer, the misalignment error, the flexural deflection angular, and its angular rate as the state of the filter:

(8) $$X={\left[\begin{array}{ccc} \begin{array}{ccc} {\left(\varphi^{n}\right)}^{T} & {\left(\delta V_{e}^{n}\right)}^{T} & {\left(\varepsilon_{b}^{bs}\right)}^{T} \end{array} & \begin{array}{cc} {\left(\nabla_{b}^{bs}\right)}^{T} & {\left(\mu^{bf}\right)}^{T} \end{array} & \begin{array}{cc} {\left(\theta^{bf}\right)}^{T} & {\left(\omega^{bf}\right)}^{T} \end{array} \end{array}\right]}^{T}$$

where $\varphi^{n}$ and $\delta V_{e}^{n}$ are the attitude error and velocity error of the slave SINS. $\varepsilon_{b}^{bs}$ and $\nabla_{b}^{bs}$ are the bias drift of gyroscope and accelerometer. $\mu^{bf}$ is the misalignment angle of the body with respect to the theoretical mounting location. $\theta^{bf}$ and $\omega^{bf}$ are the flexural deflection angle and angular rate.

Combined with Equations (2), (3) and (7), the state function of transfer alignment can be written as follows:

(9) $$\dot{X}=FX+W$$

In this equation,

$$F=[\begin{array}{ccccccc} -\left[\omega_{in}^{n}\times \right] & 0_{3\times 3} & -C_{bs}^{n} & 0_{3\times 3} & 0_{3\times 3} & 0_{3\times 3} & 0_{3\times 3}\\ \left[f^{n}\times \right] & -\left[\left(2\omega_{ie}^{e}+\omega_{en}^{n}\right)\times \right] & 0_{3\times 3} & C_{bs}^{n} & 0_{3\times 3} & 0_{3\times 3} & 0_{3\times 3}\\ 0_{3\times 3} & 0_{3\times 3} & 0_{3\times 3} & 0_{3\times 3} & 0_{3\times 3} & 0_{3\times 3} & 0_{3\times 3}\\ 0_{3\times 3} & 0_{3\times 3} & 0_{3\times 3} & 0_{3\times 3} & 0_{3\times 3} & 0_{3\times 3} & 0_{3\times 3}\\ 0_{3\times 3} & 0_{3\times 3} & 0_{3\times 3} & 0_{3\times 3} & 0_{3\times 3} & 0_{3\times 3} & 0_{3\times 3}\\ 0_{3\times 3} & 0_{3\times 3} & 0_{3\times 3} & 0_{3\times 3} & 0_{3\times 3} & 0_{3\times 3} & I_{3\times 3}\\ 0_{3\times 3} & 0_{3\times 3} & 0_{3\times 3} & 0_{3\times 3} & 0_{3\times 3} & A_{1} & A_{2} \end{array}],\;W={\left[\begin{array}{ccc} -C_{bs}^{n}\varepsilon_{w}^{bs} & C_{bs}^{n}\nabla_{w}^{bs} & 0_{15\times 1} \end{array}\right]}^{T},\;-\left[\omega_{in}^{n}\times \right]=\left[\begin{array}{ccc} 0 & -\left(\omega_{ie}\sin L+\frac{V_{E}}{R_{N}+h}\tan L\right) & \frac{V_{N}}{R_{M}+h}\\ \omega_{ie}\sin L+\frac{V_{E}}{R_{N}+h}\tan L & 0 & \omega_{ie}\cos L+\frac{V_{E}}{R_{N}+h}\\ -\frac{V_{N}}{R_{M}+h} & -\left(\omega_{ie}\cos L+\frac{V_{E}}{R_{N}+h}\right) & 0 \end{array}\right],\;\left[f^{n}\times \right]=\left[\begin{array}{ccc} 0 & -f_{z}^{n} & f_{y}^{n}\\ f_{z}^{n} & 0 & -f_{x}^{n}\\ -f_{y}^{n} & f_{x}^{n} & 0 \end{array}\right],\;-\left[\left(2\omega_{ie}^{e}+\omega_{en}^{n}\right)\times \right]=[\begin{array}{ccc} \frac{V_{D}}{R_{M}+h} & -2\left(\omega_{ie}\sin L+\frac{V_{E}}{R_{N}+h}\tan L\right) & \frac{V_{N}}{R_{M}+h}\\ 2\left(\omega_{ie}\sin L+\frac{V_{E}}{R_{N}+h}\tan L\right) & \frac{V_{D}}{R_{M}+h}+\frac{V_{N}}{R_{M}+h}\tan L & 2\omega_{ie}\cos L+\frac{V_{E}}{R_{N}+h}\\ -\frac{{2V}_{N}}{R_{M}+h} & -2(\omega_{ie}\cos L+\frac{V_{E}}{R_{N}+h}) & 0 \end{array}].$$

where $V_{N}$, $V_{E}$, and $V_{D}$ are the velocity of the vehicle in north, west, and down directions. L and h are the latitude and altitude of the vehicle. $R_{M}$ and $R_{N}$ are the meridian circle radius and prime vertical circle radius of the earth. $\omega_{ie}$ is the earth rotation rate. $f_{x}^{n},f_{y}^{n},f_{z}^{n}$ are the output specific force of the slave SINS. $\varepsilon_{w}^{bs}$ and $\nabla_{w}^{bs}$ are the noise of the gyroscope and accelerometer, which are supposed as the white noise.

(10) $$Z={\left[\begin{array}{cc} Z_{V} & Z_{\theta } \end{array}\right]}^{T}$$

The velocity measurement equation can be written as:

(11) $$Z_{V}=\delta V_{e,s}^{n}+V_{V}^{n}$$

where $\delta V_{e,s}^{n}$ is the velocity error of the slave SINS. $V_{V}^{n}$ is the measurement noise of velocity, $V_{V}^{n}=V_{w}^{n}-\delta V_{e,m}^{n}-\delta V_{LA}^{n}$. $V_{w}^{n}\;$is the speed caused by the vibration and flexural deflection. $\delta V_{e,m}^{n}$ is the velocity error of the main SINS. $\delta V_{LA}^{n}$ is the compensation value of the lever arm effect [15].

The attitude measurement value $Z_{\theta }$ can be obtained by matching the attitude matrixes of the main SINS and slave SINS. Considering the flexural deflection equation:

(12) $$Z_{\theta }=\varphi^{n}-C_{bs}^{n}\mu -C_{bs}^{n}\theta +v_{\theta }^{n}$$

where μ is the error angle between the main SINS and slave SINS. $v_{\theta }^{n}\;$ is the measurement noise of attitude error.

Combined with (11) and (12), the measurement equation can be written as:

(13) Z=HX+V

where

$$H=\left[\begin{array}{ccc} \begin{array}{cc} 0_{3\times 3} & I_{3\times 3} \end{array} & \begin{array}{cc} 0_{3\times 3} & 0_{3\times 3} \end{array} & \begin{array}{ccc} 0_{3\times 3} & 0_{3\times 3} & \begin{array}{ccc} 0_{3\times 3} & 0_{3\times 3} & 0_{3\times 3} \end{array} \end{array}\\ \begin{array}{cc} I_{3\times 3} & 0_{3\times 3} \end{array} & \begin{array}{cc} 0_{3\times 3} & 0_{3\times 3} \end{array} & \begin{array}{ccc} 0_{3\times 3} & 0_{3\times 3} & \begin{array}{ccc} -C_{bs}^{n} & C_{bs}^{n} & 0_{3\times 3} \end{array} \end{array} \end{array}\right]$$

$$V={\left[\begin{array}{cc} v_{V}^{n} & v_{\theta }^{n} \end{array}\right]}^{T}$$

According to Equations (9) and (13), we have the transfer alignment equation of “Velocity and Attitude” matching algorithm.

(14) $$\{\begin{array}{c} X_{k}=\Phi_{k,k-1}X_{k-1}+W_{k-1}\\ Z_{k}=H_{k}X_{k}+V_{k} \end{array}$$

where $X_{k}$ is the 21-dimension state vector. $Z_{k}$ is the 6-dimension measurement vector. $W_{k-1}$ is the 21-dimension state noise variance vector. $V_{k}$ is the 6-dimension measurement noise variance vector. $\Phi_{k,k-1}\;$ is the 21 × 21 state transfer matrix of the system.

## 3. The Improved Adaptive Incremental Kalman Transfer Alignment Algorithm

The measurement noise $V_{k}$ of the “Velocity and Attitude” matching method is mainly caused by the distortion of flexural deflection. However, the prior knowledge of $V_{k}$ may be different from a practical situation because of the change of slaved SINS location and the maneuvers of the aircraft. The state noise variance vector $W_{k-1}$ can also be different from its prior knowledge because the character of the MEMS IMU noise is easily affected by the temperature and vibration. Traditional Kalman filter is only available when the prior knowledge of the state noise and measurement noise are known and their character do not change, which may lead to large alignment error. Although conventional adaptive Kalman filter is able to estimate the system noise on-line, its estimation accuracy is still not good when the system noise changes greatly. In order to improve the accuracy of the filter, the adaptive incremental Kalman filter is proposed.

### 3.1. The Adaptive Kalman Filter (AKF)

The state equation and measurement equation of the conventional adaptive Kalman filter:

(15) $$\{\begin{array}{c} X_{k}=\Phi_{k,k-1}X_{k-1}+W_{k-1}\\ Z_{k}=H_{k}X_{k}+V_{k} \end{array}$$

where $X_{k}$ is the *n*-dimension state vector, $Z_{k}$ is the *m*-dimension measurement vector, and $W_{k}$ is the *p*-dimension state noise variance vector. $V_{k}$ is the *m*-dimension measurement noise variance vector. $\Phi_{k,k-1}$ is the *n* × *n* state transfer matrix of the system.

Supposing that $W_{k}$ and $V_{k}$ satisfy the following relations:

$$\{\begin{array}{c} E\left[W_{k}\right]=q_{k},\;E\left[W_{k}W_{j}^{T}\right]=Q_{k}\delta_{kj}\\ E\left[V_{k}\right]=r_{k},\;E\left[V_{k}V_{j}^{T}\right]=R_{k}\delta_{kj}\\ E\left[W_{k}V_{j}^{T}\right]=0 \end{array}$$

where $Q_{k}$ is *p* × *p* dimension symmetric non-negative definite matrix of variance. $R_{k}$ is *m* × *m* dimension symmetric positive definite matrix of variance. $\delta_{kj}$ is *Kronecher*-*δ* function.

The adaptive Kalman filter can be obtained by recursion. The estimated value of state vector $X_{k}$ represented by ${\hat{X}}_{k}$ at the time of $t_{k}$ is:

(16) $$\{\begin{array}{c} {\hat{X}}_{k,k-1}=\Phi_{k,k-1}{\hat{X}}_{k-1}+q_{k-1}\\ P_{k,k-1}=\Phi_{k,k-1}P_{k-1}\Phi_{k,k-1}^{T}+Q_{k-1}\\ K_{k}=P_{k,k-1}H_{k}^{T}\Omega_{k}^{-1}\\ {\hat{X}}_{k}={\hat{X}}_{k,k-1}+K_{k}\left[Z_{k}-{\hat{Z}}_{k-1}\right]\\ P_{k}=P_{k,k-1}-K_{k}\Omega_{k}K_{k}^{T} \end{array}$$

where

$$\begin{array}{c} {\hat{Z}}_{k,k-1}=H_{k}{\hat{X}}_{k,k-1}+r_{k}\\ \Omega_{k}=H_{k}P_{k,k-1}H_{k}^{T}+R_{k} \end{array}$$

Suppose $\varepsilon_{k,k-1}=Z_{k}-{\hat{Z}}_{k,k-1}$, and the mean value of system noise $q_{k}$ and its variance $\Omega_{k}$, the mean value of measurement noise $r_{k}$ and its variance $R_{k}$ are estimated using maximum verified method:

(17) $$\{\begin{array}{c} {\hat{q}}_{k-1}=\left(1-\frac{1}{k}\right){\hat{q}}_{k-2}+\frac{1}{k}\left({\hat{X}}_{k}-\Phi_{k,k-1}{\hat{X}}_{k-1}\right)\\ {\hat{Q}}_{k-1}=\left(1-\frac{1}{k}\right){\hat{Q}}_{k-2}+\frac{1}{k}\left(K_{k}\varepsilon_{k}\varepsilon_{k}^{T}K_{k}^{T}+P_{k}-\Phi_{k,k-1}P_{k-1}\Phi_{k,k-1}^{T}\right)\\ {\hat{r}}_{k}=\left(1-\frac{1}{k}\right){\hat{r}}_{k-1}+\frac{1}{k}\left(Z_{k}-H_{k}{\hat{X}}_{k,k-1}\right)\\ {\hat{R}}_{k}=\left(1-\frac{1}{k}\right){\hat{R}}_{k-1}+\frac{1}{k}\left(\varepsilon_{k}\varepsilon_{k}^{T}-H_{k}P_{k,k-1}H_{k}^{T}\right) \end{array}$$

The adaptive Kalman Filter is able to estimate the system and measurement noise. However, in practical application, such as airborne transfer alignment of SINS, the disturbance is very complex and the character of the system and measurement noise may change fast with time, which may lead to large estimation error by using the adaptive Kalman Filter. In order to improve the performance, we can apply the adaptive incremental Kalman Filter to airborne SINS transfer alignment.

### 3.2. The Adaptive Incremental Kalman Filter (AIKF)

In real flight, the change of two adjacent measurement values $Z_{k}$ and $Z_{k-1}$ is small. If we choose the incremental of two successive measurement values as the measurement value, which is represented by $\Delta Z_{k}$, the system error can be reduced. Based on this idea, we can obtain the state equation and measurement equation of the incremental Kalman filter [16,17]:

(18) $$\{\begin{array}{c} X_{k}=\Phi_{k,k-1}X_{k-1}+W_{k-1}\\ \Delta Z_{k}=H_{k}X_{k}-H_{k-1}X_{k-1}+V_{k} \end{array}$$

where $\Delta Z_{k}$ is the incremental of the *m*-dimension measurement vector, and $\Delta Z_{k}=Z_{k}-Z_{k-1}$, where $Z_{k}$ is the measurement vector. 

According to the principles of the independent incremental stochastic progress, the $\Delta Z_{k}$ and $\Delta Z_{k-1}$ are independent.

The adaptive incremental Kalman filter can be obtained by recursion. The estimate value of state vector $X_{k}$, represented by ${\hat{X}}_{k}$ at time $t_{k}$ is:

(19) $$\{\begin{array}{c} {\hat{X}}_{k,k-1}=\Phi_{k,k-1}{\hat{X}}_{k-1}+q_{k-1}\\ P_{k,k-1}=\Phi_{k,k-1}P_{k-1}\Phi_{k,k-1}^{T}+Q_{k-1}\\ K_{k}=\left(P_{k,k-1}H_{k}^{T}-\Phi_{k,k-1}P_{k-1}H_{k-1}^{T}\right)\Omega_{k}^{-1}\\ {\hat{X}}_{k}={\hat{X}}_{k,k-1}+K_{k}\left[\Delta Z_{k}-\Delta {\hat{Z}}_{k,k-1}\right]\\ P_{k}=P_{k,k-1}-K_{k}\Omega_{k}K_{k}^{T} \end{array}$$

where

$$\Delta {\hat{Z}}_{k,k-1}=H_{k}{\hat{X}}_{k,k-1}-H_{k-1}{\hat{X}}_{k-1}+r_{k}$$

$$\Omega_{k}=H_{k}P_{k,k-1}H_{k}^{T}+R_{k}-H_{k-1}P_{k-1}\Phi_{k,k-1}^{T}H_{k-1}^{T}-H_{k-1}\Phi_{k,k-1}P_{k-1}H_{k-1}^{T}+H_{k-1}P_{k-1}H_{k-1}^{T}$$

In the progress of SINS transfer alignment, the mean value of system noise $q_{k}$ and its variance $Q_{k}$, the mean value of measurement noise $r_{k}$ and its variance $R_{k}$ are all unknown time-varying parameters, which needs to be estimated using the maximum verified method.

(20) $$\{\begin{array}{c} {\hat{q}}_{k}=\frac{1}{k}\overset{k}{\underset{j=1}{\sum }}\left({\hat{X}}_{j}-\Phi_{j,j-1}{\hat{X}}_{j-1}\right)\\ {\hat{Q}}_{k}=\frac{1}{k}\overset{k}{\underset{j=1}{\sum }}\left({\hat{X}}_{j}-\Phi_{j,j-1}{\hat{X}}_{j-1}-{\hat{q}}_{k}\right){\left({\hat{X}}_{j}-\Phi_{j,j-1}{\hat{X}}_{j-1}-{\hat{q}}_{k}\right)}^{T}\\ {\hat{r}}_{k}=\frac{1}{k}\overset{k}{\underset{j=1}{\sum }}\left[\Delta Z-\left(H_{j}{\hat{X}}_{j,j-1}-H_{j-1}{\hat{X}}_{j-1}\right)\right]\\ {\hat{R}}_{k}=\frac{1}{k}\overset{k}{\underset{j=1}{\sum }}\left[\Delta Z-\left(H_{j}{\hat{X}}_{j,j-1}-H_{j-1}{\hat{X}}_{j-1}\right)-{\hat{r}}_{k}\right]{\left[\Delta Z-\left(H_{j}{\hat{X}}_{j,j-1}-H_{j-1}{\hat{X}}_{j-1}\right)-{\hat{r}}_{k}\right]}^{T} \end{array}$$

The estimation value ${\hat{Q}}_{k}$ and ${\hat{R}}_{k}$ are biased using the equation above. In order to obtain the unbiased estimation, we set the innovation of $\varepsilon_{k}=\Delta Z_{k}-\Delta Z_{k,k-1}$ and we have:

(21) $$E\left[\varepsilon_{k}\right]=0$$

(22) $$E\left[\varepsilon_{k}\varepsilon_{k}^{T}\right]=H_{k}P_{k,k-1}H_{k}^{T}+R_{k}-H_{k-1}P_{k-1}\Phi_{k,k-1}^{T}H_{k}^{T}-H_{k}\Phi_{k,k-1}P_{k-1}H_{k-1}^{T}+H_{k-1}P_{k-1}H_{k-1}^{T}$$

According to Equation (19), we have:

(23) $$\{\begin{array}{c} E\left[{\hat{q}}_{k}\right]=\frac{1}{k}\overset{k}{\underset{j=1}{\sum }}E\left(K_{j}\varepsilon_{j}+q_{j}\right)=q_{k}\\ E\left[{\hat{r}}_{k}\right]=\frac{1}{k}\overset{k}{\underset{j=1}{\sum }}E\left(\varepsilon_{j}+r_{j}\right)=r_{k} \end{array}$$

Then we can see that the estimation of the mean value is unbiased. Also, we have:

(24) $$\begin{array}{l} E\left[{\hat{R}}_{k}\right]=\frac{1}{k}\sum_{j=1}^{k}E\left[\varepsilon_{j}\varepsilon_{j}^{T}\right]\\ \;=\frac{1}{k}\sum_{j=1}^{k}(H_{j}P_{j,j-1}H_{j}^{T}-H_{j-1}P_{j-1}\Phi_{j,j-1}^{T}H_{j}^{T}\\ \;-H_{j}\Phi_{j,j-1}P_{j-1}H_{j-1}^{T}+H_{j-1}P_{j-1}H_{j-1}^{T})+R_{k} \end{array}$$

So, the ${\hat{R}}_{k}$ is biased. Then we introduce the suboptimal unbiased maximum a posteriori (MAP) estimator ${\hat{R}}_{k}$:

(25) $$\begin{array}{c} {\hat{R}}_{k}=\frac{1}{k}\sum_{j=1}^{k}\left(\varepsilon_{j}\varepsilon_{j}^{T}-\left(\Omega_{j}-R_{j}\right)\right)\\ =\frac{1}{k}\sum_{j=1}^{k}\left(\varepsilon_{j}\varepsilon_{j}^{T}-\left(H_{j}P_{j,j-1}H_{j}^{T}-H_{j-1}P_{j-1}\Phi_{j,j-1}^{T}H_{j}^{T}-H_{j}\Phi_{j,j-1}P_{j-1}H_{j-1}^{T}+H_{j-1}P_{j-1}H_{j-1}^{T}\right)\right) \end{array}$$

Similarly, we have:

(26) $$E\left[{\hat{Q}}_{k}\right]=\frac{1}{k}\overset{k}{\underset{j=1}{\sum }}\left(\Phi_{j,j-1}P_{j-1}\Phi_{j,j-1}^{T}-P_{j}\right)+Q_{k}$$

(27) $${\hat{Q}}_{k}=\frac{1}{k}\overset{k}{\underset{j=1}{\sum }}\left(K_{j}\varepsilon_{j}\varepsilon_{j}^{T}K_{j}^{T}+P_{j}-\Phi_{j,j-1}P_{j-1}\Phi_{j,j-1}^{T}\right)$$

The recurrence formulas of adaptive incremental Kalman filter together with the suboptimal unbiased MAP estimator are:

(28) $$\{\begin{array}{c} {\hat{q}}_{k}=\left(1-\frac{1}{k}\right){\hat{q}}_{k-1}+\frac{1}{k}\left({\hat{X}}_{k}-\Phi_{k,k-1}{\hat{X}}_{k-1}\right)\\ {\hat{Q}}_{k}=\left(1-\frac{1}{k}\right){\hat{Q}}_{k-1}+\frac{1}{k}\left(K_{k}\varepsilon_{k}\varepsilon_{k}^{T}K_{k}^{T}+P_{k}-\Phi_{k,k-1}P_{k-1}\Phi_{k,k-1}^{T}\right)\\ {\hat{r}}_{k}=\left(1-\frac{1}{k}\right){\hat{r}}_{k-1}+\frac{1}{k}\left[\Delta Z_{k}-\left(H_{k}{\hat{X}}_{k,k-1}-H_{k-1}{\hat{X}}_{k-1}\right)\right]\\ {\hat{R}}_{k}=\left(1-\frac{1}{k}\right){\hat{R}}_{k-1}\\ +\frac{1}{k}\left[\varepsilon_{k}\varepsilon_{k}^{T}-\left(H_{k}P_{k,k-1}H_{k}^{T}-H_{k-1}P_{k-1}\Phi_{k,k-1}^{T}H_{k}^{T}-H_{k}\Phi_{k,k-1}P_{k-1}H_{k-1}^{T}+H_{k-1}P_{k-1}H_{k-1}^{T}\right)\right] \end{array}$$

where $\varepsilon_{k}=\Delta Z_{k}-\left(H_{k}{\hat{X}}_{k,k-1}-H_{k-1}{\hat{X}}_{k-1}\right)-{\hat{r}}_{k-1}$.

When the noise parameters are unknown and time-varying [18,19], the estimator is:

(29) $$\{\begin{array}{c} {\hat{q}}_{k}=\left(1-d_{k-1}\right){\hat{q}}_{k-1}+d_{k-1}\left({\hat{X}}_{k}-\Phi_{k,k-1}{\hat{X}}_{k-1}\right)\\ {\hat{Q}}_{k}=\left(1-d_{k-1}\right){\hat{Q}}_{k-1}+d_{k-1}\left(K_{k}\varepsilon_{k}\varepsilon_{k}^{T}K_{k}^{T}+P_{k}-\Phi_{k,k-1}P_{k-1}\Phi_{k,k-1}^{T}\right)\\ {\hat{r}}_{k}=\left(1-d_{k-1}\right){\hat{r}}_{k-1}+d_{k-1}\left[\Delta Z_{k}-\left(H_{k}{\hat{X}}_{k,k-1}-H_{k-1}{\hat{X}}_{k-1}\right)\right]\\ {\hat{R}}_{k}=\left(1-d_{k-1}\right){\hat{R}}_{k-1}\\ +d_{k-1}\left[\varepsilon_{k}\varepsilon_{k}^{T}-\left(H_{k}P_{k,k-1}H_{k}^{T}-H_{k-1}P_{k-1}\Phi_{k,k-1}^{T}H_{k}^{T}-H_{k}\Phi_{k,k-1}P_{k-1}H_{k-1}^{T}+H_{k-1}P_{k-1}H_{k-1}^{T}\right)\right] \end{array}$$

where $d_{k-1}=\frac{1-b}{1-b^{k}}$, 0<b<1 , *b* is the forgetting factor. Usually 0.95<b<0.995. When b→1, $d_{k-1}\to \left(1/t\right)$, which is in accord with the Sage and Husa adaptive algorithm. The forgetting factor should be adjusted according to the character of the noise. If the frequency band of the noise is low, the value should be close to 1, otherwise the value should be reduced. The forgetting factor *b* can control the memory length of the filter to strengthen the estimation of the lately measurement data and reduce percentage of the old data.

### 3.3. Comparison of AKF and AIKF

**Figure 2:**
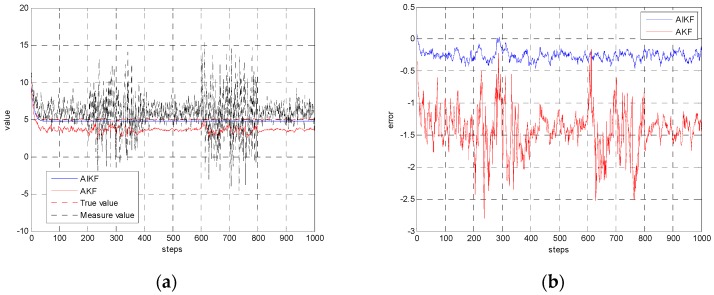
Comparison of AKF and AIKF. (**a**) Estimation result of AIKF and AKF; (**b**) Estimation error of AIKF and AKF.

To compare the estimation accuracy, the two methods of AKF and AIKF are simulated under the same condition. The results are presented as Figure 2. In Figure 2, we compare the performance of AKF and AIKF. During the steps 200~400 and steps 600~800, the measurement noise becomes worse abruptly. We can apparently see that AIKF has better estimation accuracy. The estimation error of AIKF is less than 0.5 while the error of AKF can be larger than 2.5. Besides, we can see that the estimation value of AIKF is still very smooth in the whole progress when the measurement noise changes. On the contrary, AKF is more easily affected by the disturbance of the measurement noise.

To compare the two methods in a more comprehensive way, the calculation amount of the different filtering algorithms are also considered, as is shown in Table 1.

In Table 1, we list the calculation amount of AIKF, AKF, and KF. The calculation amount is evaluated by the floating-point operations (flops), which is defined as the operation of adding, decreasing, multiplying, or dividing between two floating numbers. Supposing the dimension of the state vector is *n* and the measurement vector dimension is *m*, we can calculate the total flops of each filter algorithm. In SINS transfer alignment, the state vector *n* is 21 and the measurement vector *m* is 6. Then the total flops of AIKF, AKF, and KF are 177,300, 109,731, and 74,457, respectively. AIKF and AKF are more complex than KF because of the on-line noise estimation part. AIKF increases 61.6% calculation amount compared to AKF. However, the formulas of AIKF are still simple enough for practical application.

From the above comparison of conditional AKF and AIKF, we can obviously see that AIKF has better estimation accuracy, which is also more robust to the disturbance of system noise compared with traditional AKF. Though AIKF has a larger calculation amount, the formulas of AIKF are still simple enough to satisfy the real-time requirement in practical application.

## 4. Simulation Test and Results

**Figure 3:**
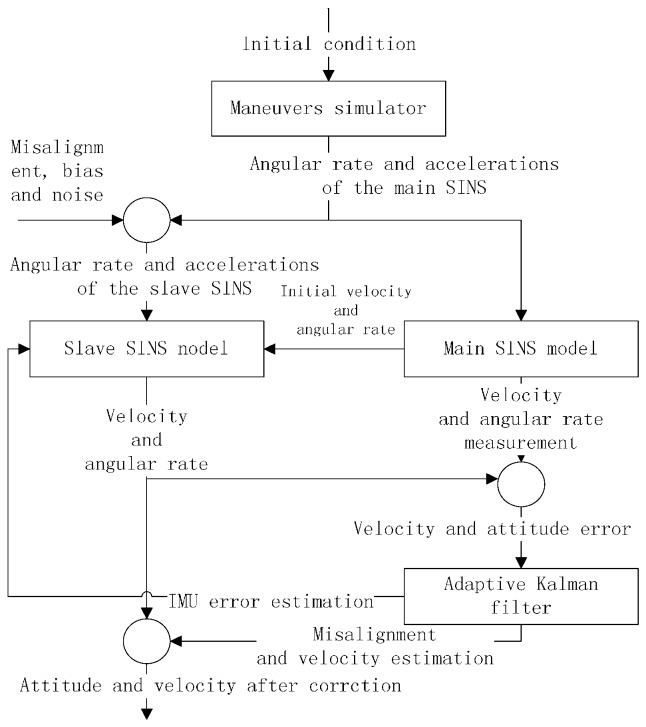
Schematic diagram of the transfer alignment algorithm.

In order to test the validity and accuracy of this algorithm, a simulation test is designed using MATLAB, which is shown in Figure 3.

Suppose the aircraft is moving straightly at 180 m/s and the yaw is −30°. During the flight, the aircraft makes two 20° swing maneuvers at the 10th second and 33rd second, respectively. In the meantime, the dynamic flexural deflection variance is set to 6′, 10′, and 7′, respectively. The correlation time parameters are 0.5, 0.4, and 10 s, respectively. The parameters of the MEMS IMU are shown in Table 2.

**Figure 4:**
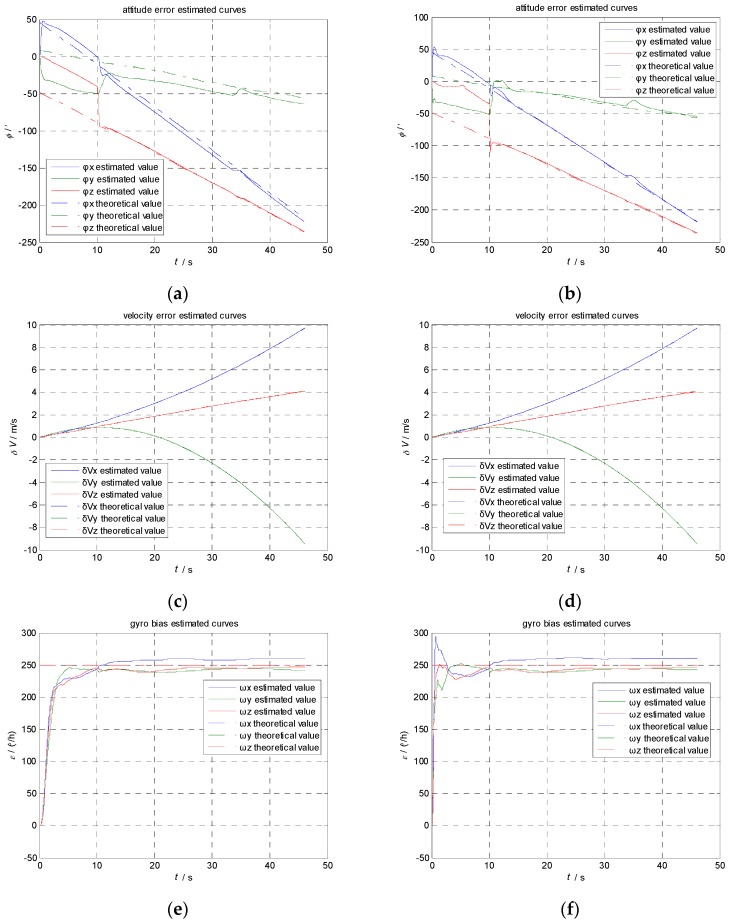

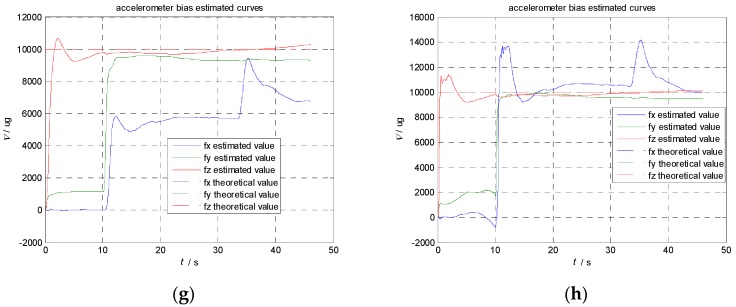
Comparison of estimation result using KF and AIKF. (**a**) Estimation of attitude error using KF; (**b**) Estimation of attitude error using AIKF; (**c**) Estimation of velocity error using KF; (**d**) Estimation of velocity error using AIKF; (**e**) Estimation of gyroscope bias using KF; (**f**) Estimation of gyroscope bias using AIKF; (**g**) Estimation of accelerometer bias using KF; (**h**) Estimation of accelerometer bias using AIKF.

**Figure 5:**
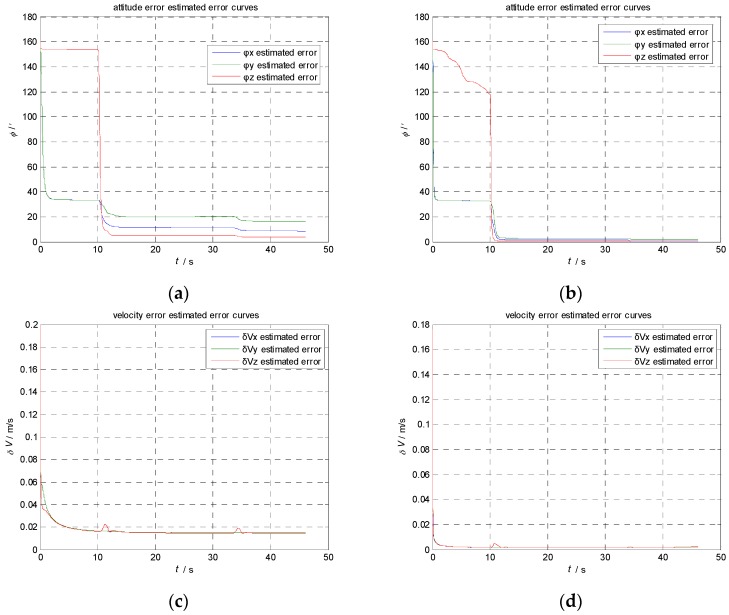

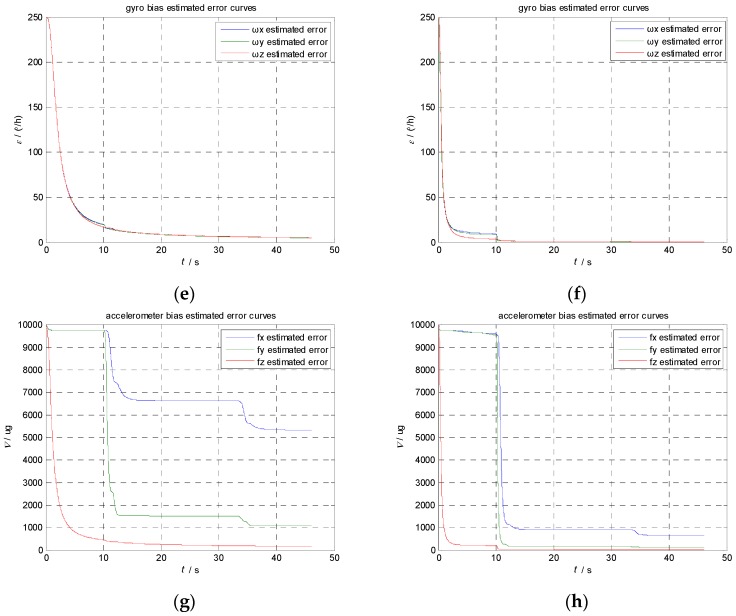
Comparison of estimation error result using KF and AIKF. (**a**) Estimation error of attitude error using KF; (**b**) Estimation error of attitude error using AIKF; (**c**) Estimation error of velocity error using KF; (**d**) Estimation error of velocity error using AIKF; (**e**) Estimation error of gyroscope bias using KF; (**f**) Estimation error of gyroscope bias using AIKF; (**g**) Estimation error of accelerometer bias using KF; (**h**) Estimation error of accelerometer bias using AIKF.

The aircraft attitude, velocity, the flexural deflection angle and its angular rate are generated by the maneuvers simulator. The transfer alignment progresses are calculated using KF and AIKF, respectively. The results are shown in Figure 4 and Figure 5.

Comparing the Figure 5g with Figure 5h, we can see the estimation error of accelerometer bias has been reduced to less than 1 mg rapidly during the first swing maneuvers using AIKF, while the estimation of accelerometer bias is much slower and its error is still around 7 mg using KF.

**Figure 6:**
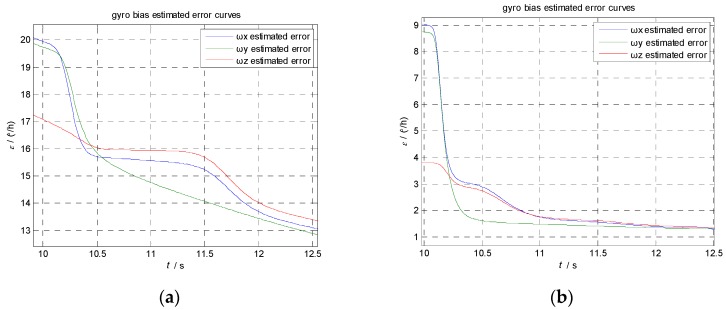
Part of the estimation error result using KF and AIKF. (**a**) Estimation error of gyroscope bias using KF; (**b**) Estimation error of gyroscope bias using AIK.

Comparing Figure 5e with Figure 5f, we can see the estimation error of gyroscope bias has been reduced to 20 °/h within 2 s using AIKF, while KF takes 10 s. If we magnify the error curve of the estimation error, as we show in Figure 6, we can see that the estimation error of gyroscope bias has been reduced from 20°/h to 13°/h in 2.5 s using KF while the AIKF reduced the error from 9°/h to 1.5°/h. The final accuracy is 5°/h by KF and 0.6°/h by AIKF, respectively. The AIKF costs less convergence time and has much better accuracy than KF.

In Table 3 and Table 4, we can see that the estimation error of gyroscope bias is less than 1°/h, the accelerometer bias is less than 1 mg, the attitude error is less than 2.5′ and the velocity error is less than 0.002 m/s. The forgetting factor *b* can control the memory length of the filter. The old knowledge of measurement data can be forgotten so that the disturbance of new measurement data will not affect the filter. So AIKF can achieve better accuracy, especially in a complicated environment. The AIKF has almost five times better accuracy than KF, which means AIKF can satisfy the requirement of airborne transfer alignment. 

## 5. Conclusions

In this paper, we develop the transfer alignment model based on the “Velocity and Attitude” matching method and the flexural deflection model. An improved AIKF algorithm is proposed to solve the problem that the state noise and measurement noise parameters cannot be accurately measured. Also, the methods of conditional AKF and AIKF are compared in terms of estimation accuracy and calculation amount, AIKF has better estimation accuracy and is more robust to the disturbance of system noise compared with traditional AKF. Though AIKF has a larger calculation amount, it can still satisfy the real-time requirement in practical application. A simulation system is designed to compare AIKF with KF. The results show that the estimation error of the gyroscope bias is less than 1°/h and the estimation error of the accelerometer bias is less than 1 mg by using AIKF, which is five times better than KF and takes less alignment time. This method can estimate the bias of the IMU, initial attitude, and velocity of SINS in a short time.

## Figures and Tables

**Table 1:** Calculation amount of each algorithm.

		AIKF	AKF	KF
Noise estimator	${\hat{q}}_{k}$	2*n*^2^ + 3*n*	2*n*^2^ + 3*n*	-
	${\hat{Q}}_{k}$	2*n*^2^ + *m*^2^ + 2*nm*^2^ − *nm* + 2*n*^2^*m* + 4*n*^3^	4*n*^3^ + 2*n*^2^*m* + 2*n*^2^ + 3*mn* − *n*	-
	${\hat{r}}_{k}$	4*mn* + 3*m*	2*mn* + 3*m*	-
	${\hat{R}}_{k}$	12*mn*^2^ + 8*m*^2^*n* + 3*m*^2^ − 6*mn*	4*m*^2^ + 2*mn*^2^ + 2*m*^2^*n* − *mn*	-
Prediction update	${\hat{X}}_{k,k-1}$	2*n*^2^	2*n*^2^	2*n*^2^
	*P_k_*_,*k−*1_	4*n*^3^ − *n*^2^	4*n*^3^ − *n*^2^	4*n*^3^−*n*^2^
Measurement update	${\hat{X}}_{k}$	10*mn* + 2*m* − *n*	4*mn* + *m*	*nm + n + m*
	*P_k_*	8*mn*^2^ + 6*m*^2^*n*-*m*^2^ − 4*mn*	4*mn*^2^ + 4*m*^2^*n* − 2*mn*	2*mn*^2^ + 2*n*^3^ − *n*^2^
	*K_k_*	4*n*^2^*m* + 2*n*^3^ + 2*nm*^2^ − *n*^2^ − 2*nm + m*^3^	2*mn*^2^ + 2*m*^2^*n* − 2*mn* + *m*^3^	4*n*^2^*m* + 4*nm*^2^ − 3*nm + m*^3^
In total		4*n*^2^ + 2*n* + 10*n*^3^ + 5*m* + 3*m*^2^ + 18*nm*^2^ + *nm* + 26*n*^2^*m* + *m*^3^	8*n*^3^ + 10*n*^2^*m* + 5*n*^2^ + 4*mn* + 2*n* + 4*m* + 4*m*^2^ + 8*m*^2^*n* + *m*^3^	6*n*^3^ + 6*mn*^2^ + 4*nm*^2^ − 2*nm* + *m*^3^ + *m* *+ n*
*n* = 21, *m*= 6		177,300	109,731	74,457

**Table 2:** The parameters of the MEMS IMU.

	Gyro	Accelerometer
**Bias drift**	250 °/h	10 mg
**Noise**	0.5 °/$\sqrt{h}$	1 mg/$\sqrt{\mathrm{Hz}}$

**Table 3:** Bias estimation result of two algorithms.

Algorithm	Estimation Error of Gyroscope Bias/(°/h)			Estimation Error of Accelerometer Bias/(mg)		
	x-Axis	y-Axis	z-Axis	x-Axis	y-Axis	z-Axis
**KF**	6.94	6.71	7.00	6.18	1.36	0.21
**AIKF**	0.81	0.81	0.73	0.81	0.14	0.02

**Table 4:** Main functional specification of MEMS gyroscope.

Algorithm	Estimation Error of Attitude Error/(′)			Estimation Error of Velocity Error /(m/s)		
	x-Axis	Y-Axis	z-Axis	x-Axis	y-Axis	z-Axis
**KF**	10.42	18.81	4.77	0.0151	0.0151	0.0147
**AIKF**	1.33	2.46	0.48	0.0016	0.0015	0.0015
